# Can Haglund’s Syndrome Be Misdiagnosed as Low Back Pain? Findings from a Case Report in Physical Therapy Direct Access

**DOI:** 10.3390/healthcare9050508

**Published:** 2021-04-28

**Authors:** Filippo Maselli, Lorenzo Storari, Valerio Barbari, Giacomo Rossettini, Firas Mourad, Mattia Salomon, Mattia Bisconti, Fabrizio Brindisino, Marco Testa

**Affiliations:** 1 Department of Neurosciences, Rehabilitation, Ophthalmology, Genetic and Maternal Infantile Sciences (DINOGMI), University of Genova—Campus of Savona, 17100 Savona, Italy; lorenzo.storari93@gmail.com (L.S.); ft.valeriobarbari@gmail.com (V.B.); giacomo.rossettini@gmail.com (G.R.); marco.testa@unige.it (M.T.); 2 Sovrintendenza Sanitaria Regionale Puglia INAIL, 70126 Bari, Italy; 3 Department of Clinical Science and Traslational Medicine, University of Roma “Tor Vergata”, 00133 Roma, Italy; 50firas@gmail.com (F.M.); salomon.mattia@gmail.com (M.S.); 4 Department of Medicine and Health Science “Vincenzo Tiberio”, University of Molise C/da Tappino c/o Cardarelli Hospital, 86100 Campobasso, Italy; biscontimattia@gmail.com (M.B.); fabrindi@gmail.com (F.B.)

**Keywords:** case report, differential diagnosis, direct access, Haglund syndrome, physical therapy, referral and consultation

## Abstract

**Background:** Haglund’s syndrome (HS) is a painful condition that is caused by an exostosis of the posterior superior part of the calcaneus coupled with Achilles tendinopathy and retrocalcaneal bursitis. Both for the proper musculoskeletal assessment and for the differential diagnosis process of possible concurrent diseases deriving from other anatomical areas, the diagnosis of HS is still a challenge. **Case Presentation:** A 41-year-old male amateur runner was diagnosed and treated for low back pain and referred leg pain by his general practitioner. Due to ineffective results, he self-presented to a physical therapist (PT) with intense right heel pain, radiating up to the leg and to the lumbopelvic region. **Results:** The PT’s examination and interview relating to the sports activities led to the correct diagnosis and a proper orthopedic referral. At the one-year follow-up, the patient reported regular pain-free marathon running. **Discussion:** This case report highlights the central role of PTs working in direct access environments as primary care healthcare professionals for the management of musculoskeletal diseases, and their abilities in identifying patients with suspected pathologic conditions that may need referral for imaging, medical assessment or surgical intervention.

## 1. Introduction

Running is one of the most popular sports for adults [1] and improves several indices of health. [2,3] Unfortunately, running is also associated with many musculoskeletal (MSK) running-related injuries [4], with a reported incidence ranging between 19% to 79% [4,5], mostly involving foot and ankle joints [5], including Haglund’s syndrome (HS). HS is a common painful MSK condition in adults caused by an exostosis in the posterior superior aspect of the calcaneus, associated with a chronic insertional Achilles tendinopathy and retrocalcaneal bursitis, caused by altered foot or ankle joint biomechanics, unsuitable footwear or chronic load stress [5,6,7]. Typically, the diagnostic process is performed through clinical assessment and the management is predominantly conservative, with surgery needed only for nonrespondent patients [6,8,9]. Recent literature findings have highlighted that physical therapy seems to be more effective, safer and cost-effective than the usual general practitioner (GP)-led care [10,11] in the management of the most burdensome MSK disorders, especially when provided in direct access (DA). DA to a physical therapist (PT) indicates the possibility of accessing this service for both assessment and treatment, without the medical prescription required by national legislation [12]. In contrast to what is happening in other countries [13,14], Italian PTs are not allowed to make a diagnosis or prescribe imaging tests, and they can only evaluate and manage mild MSK conditions, with the charge of referring for medical attention any specific conditions beyond their scope of practice [12]. We reported the pathway of care of a marathon runner who was misdiagnosed with sciatica from his GP. Because of the inconclusive results, he decided to seek a PT who recognized the HS and referred him to an orthopedic surgeon. This is the first case comparing GP-led care with DA to a PT, the more frequent occurrence of which could exert a positive impact on the healthcare service.

## 2. Case Presentation

**Figure 1:**
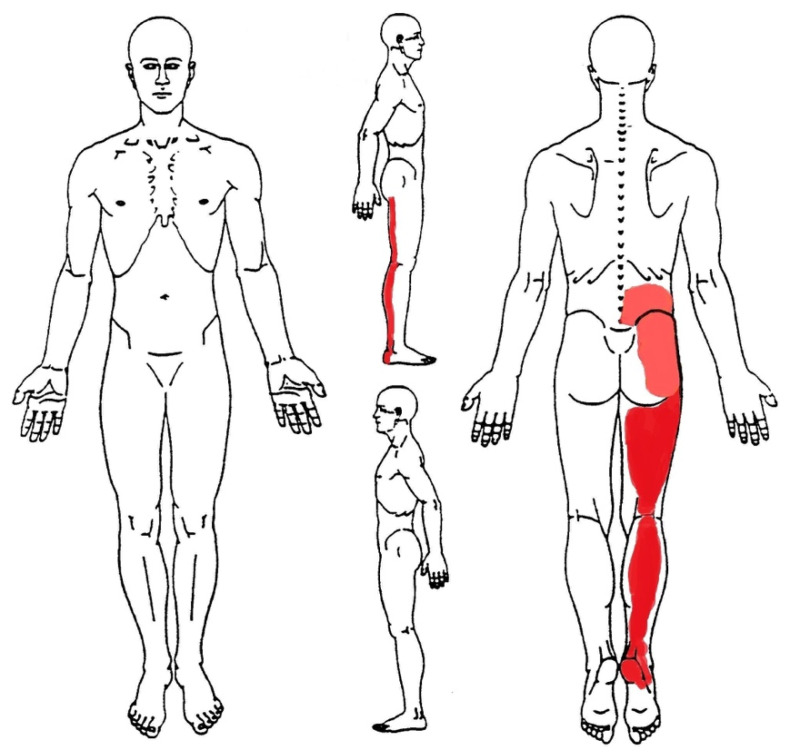
Body chart: Bright red indicates the most painful body areas; pale red indicates the mild painful body areas.

A 41-year-old male amateur runner, industrial worker, presented to his GP complaining of deep and burning pain wrapping all his right lower limb, from the calcaneus to the lumbopelvic region (see the body chart in Figure 1). Pain was stated with numeric pain rating (NPRS) scale at 8/10 in the heel, 6/10 in the calf, and 3/10 in the thigh and lower back. The patient had 15 years of experience of marathon races and usually trained 6 days per week (50 km/week). The pain started 6 months ago from his right heel (NPRS 3/10), and then radiated up to the lower back. Subsequently, due to the progression of mileage from 50 to 80 km/week in view of a marathon race, his symptoms had further worsened. He denied smoking or drinking habits. After an unremarkable physical examination, the GP diagnosed a low back pain with referred leg pain, prescribing him a painkiller drug (600 mg dose of ibuprofen for 2 times/day for 10 days). However, the symptoms did not improve, thus the GP referred him to the physical therapy service with a prescription of electrotherapy and sports massage (3 times/week for 4 weeks) for the lower back and right lower limb muscles. Nevertheless, the symptoms further worsened in the meantime, and at the end of the treatments the patient self-presented to a PT.

## 3. Results

### 3.1. Differential Diagnosis

The patient denied any trauma, unexplained weight loss, history of malignancy or any other constitutional symptoms. After a careful history taking, a thorough MSK evaluation was performed. The diagnostic criteria of low back pain with referred leg pain (characteristics of the onset and type of pain, presence of antalgic postures of the lower back, cranial to caudal progression of the symptoms, neurological signs, positive lower limb nerve tension tests), were not found during the examination of the back. Therefore, the PT decided to perform a thorough examination of the lower limb.

**Figure 2:**
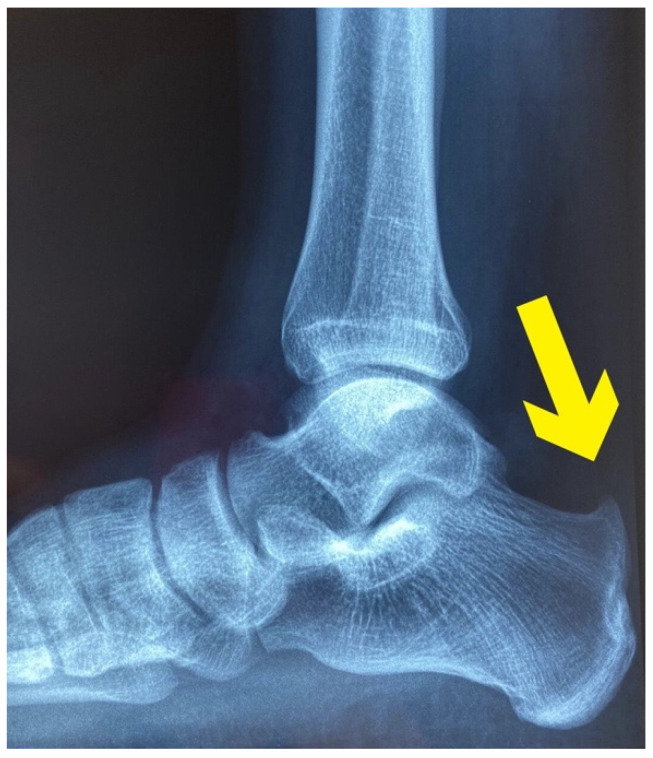
X-rays: The yellow arrow indicates an abnormal exostosis on the posterior superior aspect of the calcaneal bone.

**Figure 3:**
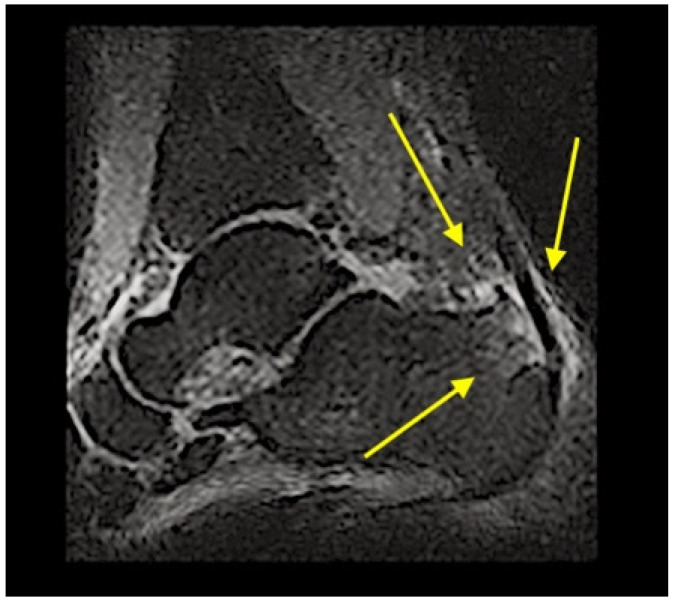
Magnetic resonance Imaging: The yellow arrows indicate: the degenerative changes in the Achilles tendon, bony oedema and the presence of retrocalcaneal bursitis, tendonitis phenomena and spongious oedema in the insertional region of Achilles tendon in the calcaneus.

**Figure 4:**
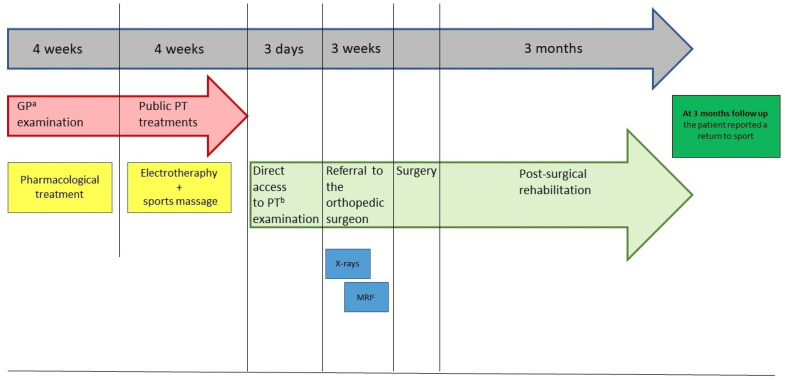
Timeline: ^a^: general practitioner; ^b^: physical therapist; ^c^: magnetic resonance imaging.

The latter revealed a prominent tubercle on the posterior superior and slightly lateral aspect of the heel. This tubercle was red, swollen and highly painful to palpation. Functional and neuromuscular tests such as heel walk and walk on toes were both possible, but the heel walk exacerbated the familiar and intense pain at the right heel (NPRS 7/10). Moreover, bilateral heel rise tests were painful (NPRS 5/10) after 20 repetitions [15], and single heel rise was extremely painful and suspended after 2 repetitions (NPRS 9/10) [16]. Manual resisted tests of the several muscles of the lower limb (plantar flexors, knee flexors, medium and gluteus maximus) pointed out unaffected strength, but palpation of biceps femoris, soleus and fibularis longus muscles were painful. Particularly, the manual pressure on the taut bands performed during the evaluation of the soleus and fibularis longus muscles reproduced the familiar pain localized in the posterior aspect of the leg and the thigh. Moreover, the pressure on the biceps femoris replicated the symptoms felt in the right lumbopelvic region. Finally, a functional questionnaire developed for Achilles tendinopathy, VISA-A questionnaire [17], was administered and it scored 45/100 points. Based on the findings of the clinical examination, the PT suspected a HS [6]. Therefore, the patient was referred to an orthopedic doctor for a detailed examination. From the assessment of the plain radiograph (Figure 2.), and the magnetic resonance imaging (Figure 3.), the orthopedist diagnosed a HS, and decided to perform a posterior ankle endoscopic calcaneoplasty and removal of the bursa. For a detailed description of the timeline management, see Figure 4.

### 3.2. Treatment

In the first month after surgery, the PT’s program (3 sessions/week) was focused on restoring ankle and foot mobility, reducing pain and swelling, and walking retraining. Passive and active ankle joint mobilizations were performed to restore the arthrokinematic movements. Calf and lower back pain were successful treated with manual digital pressure of the myofascial pain points in the soleus, fibularis longus, and biceps femoris. Plantar flexors, knee flexors, gluteus medius and maximus muscles were strengthened with isometric exercises, and then with elastic resistance. The pain-free walking was achieved through balance exercises, aerobic reconditioning with an exercise bike, and neuromuscular exercises after one month from surgery.

### 3.3. Outcome and Follow-Up

From the second postsurgical month, a progressive training of the lower limb strength and running was undertaken, with a scheduling of 2 sessions/week till the third month). The subject was evaluated on an instrumented treadmill (MyRun, Technogym, Cesena, Italy) at a running pace of 6.5 min/km, and foot strike pattern was determined analyzing slow-motion video recording. The running benchmarks were analyzed using the OPTO-JUMP NEXT software (Microgate, Bolzano, Italy). For a detailed description of the outcome measures see Table 1. At the three-month postintervention follow-up, the patient returned to a regular running training. Finally, at one-year follow-up, the patient returned to run a marathon race. 

## 4. Discussion

**Figure 5:**
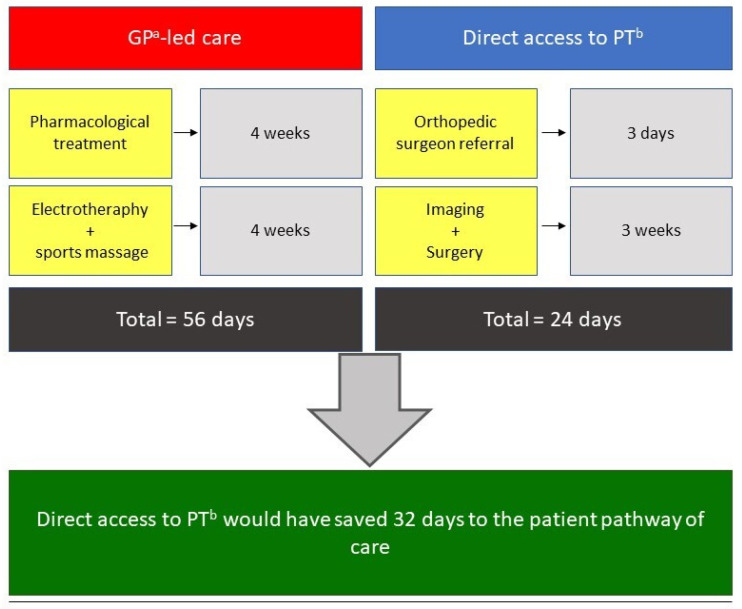
Direct Access to a PT versus GP-led care: ^a^: general practitioner; ^b^: physical therapist.

To the best of the authors’ knowledge, this is the first case report that compares the management of a common MSK disease like HS performed by a PT in DA, with the usual GP-led care. Despite the major clinical signs and symptoms of HS being clearly reported in the literature [18], and its symptom presentation may be various [19,20], a trained MSK healthcare professional should know how to recognize this type of MSK disorder, which usually causes pain or discomfort limited to the foot/Achilles tendon or posterior tibial region [21,22]. As in the present case, several scientific papers stated that experienced PTs have the knowledge needed for managing MSK conditions, even more than medical students and all physician specialists, except for orthopedists [23,24,25]. Given that the worldwide demand for MSK care is rising, and this situation is a growing challenge for GPs [26], DA to a PT could be a valid option to reduce the general practice workload and medical expenses [27]. Moreover, DA to a PT seems to offer appropriate care for MSK pain-suffering patients [10,12,28,29,30], and has produced improved functional outcomes and better cost-effectiveness ratios [29]. Indeed, patients in DA received fewer imaging investigations and a higher percentage of active treatments than passive, resulting in a lower burden of time and economical costs [31]. It is noteworthy that our comparison of the GP-led care and the DA model of care has demonstrated a potential reduction of 32 days in favor of the latter (see Figure 5), resulting in a faster pathway, even when a proper medical referral is needed. Unfortunately, even if DA has become a common practice in some countries, such as the Netherlands [13], the United States and Australia [24,32], this practice is still a gray area around the world. From the patient’s perspective, the thorough assessment and physical examination conducted by the PT and his ability to successfully identify a MSK disorder needing a proper medical referral, has demonstrated the professional skills of the PT and a comprehensive approach toward the patient’s health status. This case report supports the role of the PT in evaluating and managing MSK disorders in DA and serves a call to action to update the pathway of care of such conditions, in line with other western societies.

## 5. Conclusions

Differential diagnosis is a key element of the PT’s clinical reasoning in DA settings, even if the patients have already been assessed or treated by other healthcare professionals. In fact, applying a thorough diagnostic process, PTs in DA are able to carry out a comprehensive clinical evaluation of different MSK afflictions and to screen doubtful conditions that could indicate an appropriate medical referral.

### Learning Points

- HS is a common MSK condition that every trained healthcare professional should be able to recognize and properly manage.
- DA to a PT is a safe, cost-effective and time-saving practice for patients with MSK disorders.
- A thorough physical examination within the differential diagnosis process is mandatory in case of patients with MSK disorders.

## Figures and Tables

**Table 1:** Outcome and follow-up.

Before Surgery	Two-Month Follow-Up	Three-Month Follow-Up
NPRS: 7/10 at right heel, 6/10 from the heel to the calf, 2/10 in the lower back.	NPRS: 3/10 at right heel, 4/10 from the heel to the calf, 0/10 in the lower back.	NPRS: 0/10 throughout the body.
Bilateral heel rise tests were painful (NPRS 5/10) after 20 repetitions	---	Bilateral heel rise test: 69 repetitions
Single heel rise was extremely painful and suspended after 2 repetitions (NPRS 9/10)	---	Single heel rise test: 29 repetitions
VISA-A questionnaire: 45/100 points	---	VISA-A questionnaire: 95/100 points
---	Rearfoot strike pattern	Midfoot-strike pattern
---	Running pace was on average 6.5 min/km	Running pace was on average 5 min/km
---	Running cadence: 167 steps per minute	Running cadence: 181 steps per minute
---	Ground contact time: 277 ms	Ground contact time: 252 ms
---	Vertical oscillation: 8 cm	Vertical oscillation: 7 cm

Acronyms: NPRS: numeric pain rating scale; ms: milliseconds; cm: centimeters; VISA-A: Victorian Institute of Sports Assessment–Achilles questionnaire.

## Data Availability

Not applicable.
